# Comparison of the Biological Impact of UVA and UVB upon the Skin with Functional Proteomics and Immunohistochemistry

**DOI:** 10.3390/antiox8120569

**Published:** 2019-11-20

**Authors:** Pei-Wen Wang, Yu-Chiang Hung, Tung-Yi Lin, Jia-You Fang, Pei-Ming Yang, Mu-Hong Chen, Tai-Long Pan

**Affiliations:** 1 Department of Medical Research, China Medical University Hospital, China Medical University, Taichung 40447, Taiwan; pwwang5105@hotmail.com; 2 Department of Chinese Medicine, College of Medicine, Kaohsiung Chang Gung Memorial Hospital and Chang Gung University, Kaohsiung 83301, Taiwan; e120845@cgmh.org.tw; 3 Department of Traditional Chinese Medicine, Chang Gung Memorial Hospital, Keelung 20401, Taiwan; tungyi30@cgmh.org.tw; 4 Pharmaceutics Laboratory, Graduate Institute of Natural Products, Chang Gung University, Taoyuan 33302, Taiwan; fajy@mail.cgu.edu.tw; 5 TMU Research Center of Cancer Translational Medicine, Taipei Medical University, Taipei 11042, Taiwan; yangpm@tmu.edu.tw; 6 Graduate Institute of Cancer Biology and Drug Discovery, College of Medical Science and Technology, Taipei Medical University, Taipei 11042, Taiwan; 7 Department of Psychiatry, Taipei Veterans General Hospital, Taipei 11217, Taiwan; kremer7119@gmail.com; 8 Department of Psychiatry, College of Medicine, National Yang-Ming University, Taipei 11221, Taiwan; 9 School of Traditional Chinese Medicine, Chang Gung University, Taoyuan 33302, Taiwan; 10 Liver Research Center, Chang Gung Memorial Hospital, Taoyuan 33375, Taiwan

**Keywords:** UV radiation, skin, oxidative stress, proteomics, network analysis, immunohistochemistry

## Abstract

The skin provides protection against external stimuli; however, solar radiation, including ultraviolet A (UVA) and ultraviolet B (UVB), can result in profound influences on skin structure and function, which eventually impairs its molecular characteristics and normal physiology. In the current study, we performed proteome tools combined with an immunohistological approach on nude mouse skin to evaluate the adverse responses elicited by UVA and UVB irradiation, respectively. Our findings indicated that UVA significantly promotes oxidative damage in DNA, the breakdown of collagen fiber in the dermis, and the apoptosis of fibroblasts, which leads to inflammation. Meanwhile, UVB administration was found to enhance the carbonylation of various proteins and the proliferation of keratinocyte. Particularly, raspberry extract, which has been confirmed to have antioxidative efficacy, could effectively attenuate ultraviolet (UV) radiation-caused cell death. Network analysis also implied that UVA and UVB induce quite different responses, and that UVA results in cell death as well as inflammation mediated by caspase-3 and activator protein 1/nuclear factor kappa-light-chain-enhancer of activated B cells (AP-1/NF-κB), while UVB predominantly increases the risk of skin carcinogenesis involved with oncogenes such as p53 and c-Myc. Taken together, functional proteomics coordinated with histological experiments could allow for a high-throughput study to explore the alterations of crucial proteins and molecules linked to skin impacts subjected to UVA and UVB exposure.

## 1. Introduction

Human skin is normally exposed exclusively to wavelengths less than 294 nm, while solar ultraviolet (UV) radiation, which contains both UVA (320–400 nm) and UVB (290–320 nm), might cause various injuries to the skin [1,2]. UVB elicits alterations majorly at the epidermal level, where the large scale of UVB is uptaken. The amount of UVA in ambient light surpasses the amount of UVB by 10 to 100 times; however, UVB radiation is more energetic than UVA radiation. UVA penetrates deeper than UVB into the skin layer and mediates damage to both the epidermis and dermis. In this regard, UVA and UVB have different characteristics and induce divergent responses in the skin [3,4,5]. Currently, there is a growing need for standardization and evaluation of the biological events and molecular mechanisms aroused by UVA and UVB, which is important for clinical therapy and cosmetic application in the future.

Large amounts of proteins and molecules are altered in regard to quantity and quality during the development of skin problems caused by environmental stress, which reflects pathological abnormalities and disease progression [6,7,8,9]. Traditional analytical methods are not enough to elucidate complex biochemical processes or biological functions derived from the changes in protein profiles. Therefore, proteome methods coordinated with bioinformatics for data mining have provided a feasible tool for large-scale screening and differentially identifying protein targets that are linked to the pathogenesis and the pinpointing signaling pathways associated with UV-mediated skin damage [10,11,12]. Herein, we utilized MetaCore pathway software to comprehensively dissect the cellular pathways behind the differences in protein levels.

Numerous reports have shown that UV radiation, including UVA and UVB, significantly disturbs the redox balance in human skin cells due to the overproduction of reactive oxygen species (ROS), which impair antioxidant systems such as thioredoxin and glutathione–glutathione peroxidase [13,14,15]. Oxidative stress further results in the oxidative modifications of lipids, proteins, and DNA [16]. Moreover, oxidative damage to proteins with disrupted structures would be targeted for degradation by the cellular proteolytic systems, which in turn may enhance apoptotic events [17]. In addition, oxidative stress has been reported to play a pathogenic role in chronic inflammatory diseases; protein oxidations release inflammatory signaling molecules, leading to a visible deterioration in skin [18,19]. Thereby, the application of antioxidants is a practical strategy for UV protection of the skin. 

Protein modification caused by oxidative stress would result in the generation of carbonyl groups that can react with 2,4-dinitrophenylhydrazine (DNP) and are investigated by specific antibodies. Redox proteomics could be utilized to delineate the status in protein carbonylation [20]. Collectively, these tools provide an opportunity to study the critical proteins and molecules linked to the complicated signaling pathways of living cells and promise a feasible approach to explore the novel factors modulating cell function under different types of UV radiation.

Despite previous documents mentioning the possible pathology and mechanisms related to skin injuries caused by UVA or UVB, no effort has been made to distinguish the discrepancy in global protein expression and the biological effects between these two solar radiations. Our findings could offer new opportunities for therapeutic intervention aimed at skin protection from different kinds of UV wavelengths. 

## 2. Materials and Methods

### 2.1. Experimental Animals

Female nude mice (ICR-Foxn/nu) were purchased from Taiwan’s National Laboratory Animal Center (Taipei). The treatments to the mice were based on the Ethical Guidelines of the Animal Center, and the experimental methods were ratified by the Institutional Animal Care and Use Committee of Kaohsiung Chang Gung Memorial Hospital (2017081401). The mice were randomly divided into three groups (control (CTL), UVA, and UVB) of five mice each. A Bio-Sun system illuminator (Vilber Lourmat, Marne-la-Vallée, France) was applied to generate UVA (λ_365 nm_) and UVB (λ_312 nm_) radiation. The spectral irradiance was 10 joules/cm^2^ (J/cm^2)^ for UVA and 175 milli joules/cm^2^ (mJ/cm^2^) for UVB, respectively. UVA was irradiated on the dorsal region of the mouse on every other day for three days and UVB was applied on the mice once a day for five days. The duration of UVA and UVB exposure was 100 and 1 min [21].

### 2.2. Histological Study of the Mouse Skin

The skin samples were fixed in formaldehyde solution and sliced into 5-µm sections which were then applied with hematoxylin and eosin (H&E) and Masson’s trichrome staining for the histological examination. Immunohistochemistry evaluation of 8-OHdG, proliferating cell nuclear antigen (PCNA), Hsp27 or c-Jun (Santa Cruz Biotechnology, Dallas, TX, USA) was conducted with specimens as described in the previous manuscript [10]. The histological changes in non-consecutive and randomly chosen fields were further determined with optical microscopy (Olympus BX51, Tokyo, Japan). The quantification of the signal was performed with Image-Pro^®^ plus 4.5 (Mediacybernetics, Bethesda, MD, USA) according to the protocol described by McGinley et al. [22].

### 2.3. Terminal Deoxynucleotidyl Transferase dUTP Nick End Labeling (TUNEL) Assays

TUNEL assay was conducted with ApopTag® Plus Peroxidase In Situ Apoptosis Detection Kit (Millipore, Burlington, MA, USA). The stained and unstained cells from randomly chosen fields per slide were counted. Finally, Mayer’s hematoxylin was counterstained and the slides were observed under the microscope [23].

### 2.4. Gelatin Zymography

Smashed skin samples were gathered and extracted for analysis of gelatin zymography as previously described [24]. Briefly, gels were washed with 50 mM Tris-HCl, at pH 7.5, containing 2.5% Triton X-100 (*v*/*v*) after electrophoresis and incubated in 50 mM Tris-HCl buffer containing 5 mM CaCl_2_. Digestion was terminated and then the gels were stained with 0.5% Coomassie brilliant blue R250 followed by 10% acetic acid and 10% methanol for destaining. White bands against a blue background were found in enzyme-digested parts and the band intensity was quantified with GeneTools Image Software (Syngene, Cambridge, UK).

### 2.5. Two-Dimensional Electrophoresis (2-DE) Analysis

The skin sample stripped from the subcutaneous layer was washed by phosphate buffered saline (PBS), homogenized, and soaked in extraction buffer (tissue/solution ratio: 40 mg skin sample in 1 mL buffer containing 7 M urea, 2 M thiourea, 4% 3-(3-cholamidopropyl) dimethylammoniopropane sulfonate (CHAPS), 65 mM dithiothreitol (DTT), 1 mM phenylmethanesulfonyl fluoride (PMSF). Then, the homogenate was centrifuged at 10,000× *g* for 20 min (KUBOTA, Osaka, Japan). The Bradford Protein Assay Kit (AMRESCO) was applied to determine the concentration of the supernatant. Then, 200 μg protein was solubilized in an immobilized pH gradient (IPG) buffer containing 7 M urea, 2 M thiourea, 4% CHAPS, 65 mM DTT, and 1% IPG buffer to a final volume of 350 μL. The Immobiline Drystrip (18 cm, pH 4–7 IPG linear strip, GE Healthcare, Göteborg, Sweden) was used to separate the proteins on the IPGphor III System for the first dimension. The IPG strips were equilibrated in a solution containing 50 mM Tris-HCl (pH 8.8), 6 M urea, 2% SDS, 30% glycerol, and 2% DTT followed by exposure of the same solution except that DTT was substituted with 2.5% iodoacetamide. The 2-DE was conducted with 10% acrylamide gels (Bio-Rad, Hercules, CA, USA) and then visualized by silver staining. The Prodigy SameSpots software (Nonlinear Dynamics, Newcastle, UK) was performed to quantify the protein spots and each spot intensity volume (%) was detected after background subtraction and total spot volume normalization. More than 2.0-fold changes at 95% confidence interval (*p* < 0.05) were considered as statistically significant [10,25].

### 2.6. Determination of Protein Carbonyls with DNP Immunostaining

The IPG strips were immersed in 2N HCl with 10 mM DNP (2,4-dinitrophenylhydrazine, Sigma, St. Louis, MO, USA) and neutralized with 2 M Tris–base/30.0% glycerol. The IPG strips were then prepared for molecular weight-based separation of DNP-modified proteins by SDS-PAGE gel, followed by transferring to a polyvinylidene difluoride (PVDF) membrane as described previously [26]. The PVDF membranes were then incubated overnight for immunostaining with the solution containing the anti-DNP IgG antibody (Sigma) in the tris-buffered saline Tween-20 (TBST) with 5.0% non-fat milk. The blots were next incubated with the goat anti-rabbit immunoglobulin G/ horseradish peroxidase (IgG/HRP) conjugate and the enhanced chemiluminescence kit (Immobilon Western Chemiluminescent HRP substrate, Millipore, Bedford, MA, USA) was applied for signal detection.

### 2.7. In-Gel Digestion and Mass Spectrometric Analysis

Targeted protein spots were excised from the gels and digested with trypsin as previously described [27]. The tryptic peptides were acidified with 0.5% trifluoroacetic acid (TFA) and loaded onto an MTP AnchorChip™ 600/384 TF (Bruker-Daltonik, Bremen, Germany) after digestion. MS analysis was conducted using the Ultraflex™ MALDI-TOF mass spectrometer (Bruker-Daltonik). Monoisotopic peptide masses were assigned and utilized for database searches with the MASCOT search engine (version 2.2.04, Matrix Science, London, UK). Search parameters were enacted as follows: a maximum allowed peptide mass error of 50 ppm, and consideration of one incomplete cleavage per peptide.

### 2.8. Network Analysis with MetaCore™

MetaCore™ software (version 5.2 build 17389, GeneGo, St. Joseph, MI, USA) was used to explore related ontological classes and corresponding pathways which were denoted among the proteins revealed by the 2-DE and peptide mass fingerprint [27].

### 2.9. Western Blot Experiments

The skin tissue was homogenized, and the protein was isolated with 1× cell lysis buffer (Cell Signaling, Danvers, MA, USA). Then the concentration was measured with the Bradford Protein Assay Kit (AMRESCO). Proteins were separated with 10% SDS-PAGE (W × L: 7.2 cm × 8.6 cm) and transferred to a PVDF membrane. The specific antibodies for c-Myc and glyceraldehyde 3-phosphate dehydrogenase (GAPDH) (Santa Cruz Biotech.) were applied and enhanced chemiluminescence was used for signal detection. The result was quantified with GeneTools software (Syngene, Cambridge, UK). GADPH was used as the internal control.

### 2.10. Evaluation of Cell Viability by MTT Analysis

HaCaT cells (5 × 10^4^) were seeded in 24-well plates for 24 h. UVA and UVB radiation (24 or 0.24 J/cm^2^) were exposed to the cells after treating with raspberry extract (0.5 mg/mL) and incubated for 48 h [25]. Isopropanol solution mixed with tetrazolium salt was then added to the wells and incubated for additional 4 h at 37 °C. The optical density of the dissolved material was measured spectrophotometrically at 570 nm. 

### 2.11. Statistical Analysis

Statistical calculation was performed using the ANOVA test and the post hoc Newman–Keuls test was applied to determine the individual differences between the groups with Prism software (v5.0, Prism GraphPad, San Diego, CA, USA). Differences were considered significant at * *p* < 0.05, ** *p* < 0.01, and *** *p* < 0.001. Data was confirmed through three technical repetitions.

## 3. Results

### 3.1. Histological Assessment

**Figure 1:**
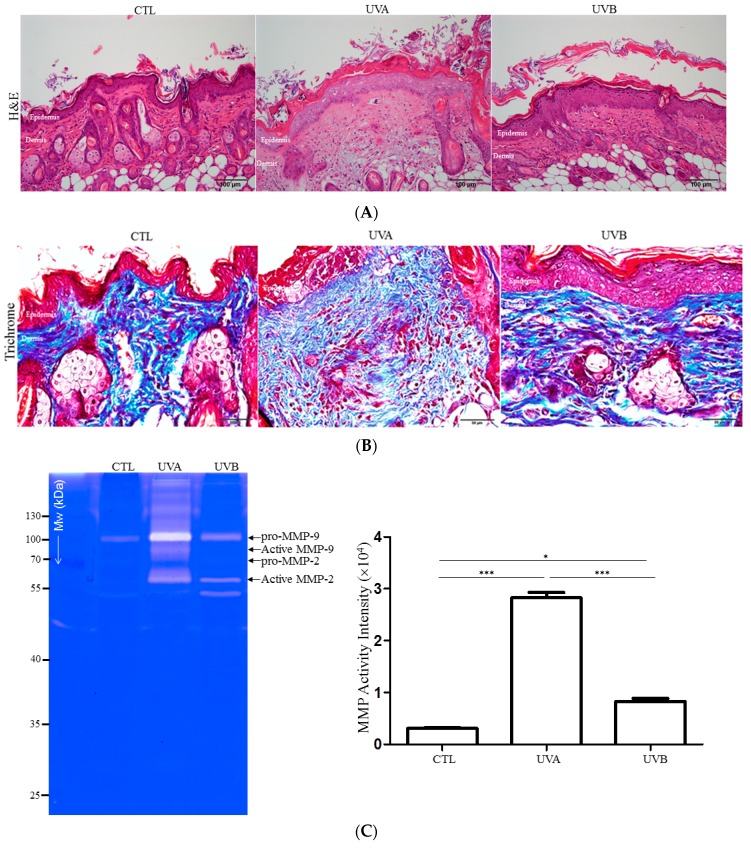
The histologic examination and matrix metalloproteinase (MMP) secretion of the mouse skin exposed to UVA or UVB. (**A**) Histological analysis and assessment of nude mouse skin with hematoxylin and eosin (H&E) staining from control (CTL), UVA-treated, and UVB-exposed samples. Original magnification: 100×. (**B**) Evaluation of the structure and amount of collagen via trichrome-staining. Original magnification: 100×. (**C**) MMP expression was assayed by gelatin zymography of control, UVA-, and UVB-applied skin. Quantified data were the mean ± SD of three independent experiments. (* *p* < 0.05; *** *p* < 0.001).

A histopathological analysis of the mouse skin samples exposed to UVA or UVB was performed to evaluate the possible damage induced by different types of solar radiation. The control skin showed an intact structure and constitution of full-thickness skin with compact keratinized cell layers, while a remarkable proliferation of epidermal cells was observed in the UVB-exposed skin. Meanwhile, increased infiltration of leukocytes and the breakdown of dermis collagen were demonstrated in the UVA-irradiated skin (Figure 1A). Again, Masson’s trichrome stain indicated that the collagen fiber was irregular, loose, curled in contour, and had the formation of a great number of peptide fragments under UVA exposure, whereas the UVB application was identified as having a much more compressed collagen pattern. The control skin was characterized by having a thin reticulated fiber structure (Figure 1B).

Moreover, the effect of solar radiation on matrix metalloproteinase (MMP) secretion was studied on UV-damaged skin. An obvious upregulation of MMP expression was identified in the UVA-irradiated skin, whereas a moderately increased level of MMP was present in the UVB-irradiated skin with respect to the control sample (Figure 1C). These results provided evidence that UVA strongly enhances the MMP elevation and degradation of dermal collagen, resulting in the integrity of the dermis being compromised and the reparative response becoming flawed.

### 3.2. Effects of UVA and UVB Exposure in the Generation of Oxidative Stress

**Figure 2:**
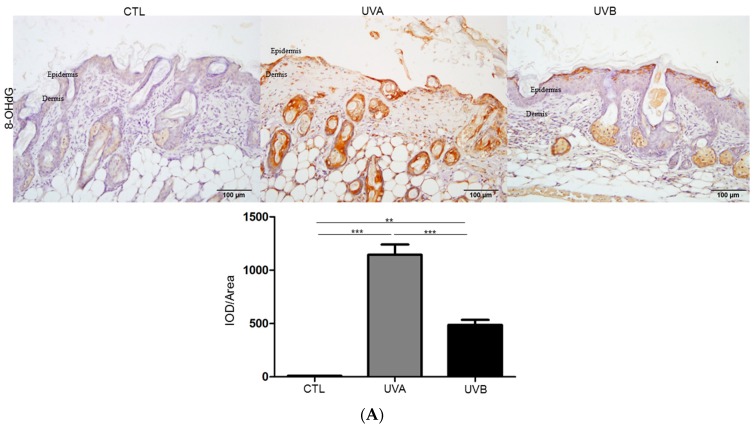

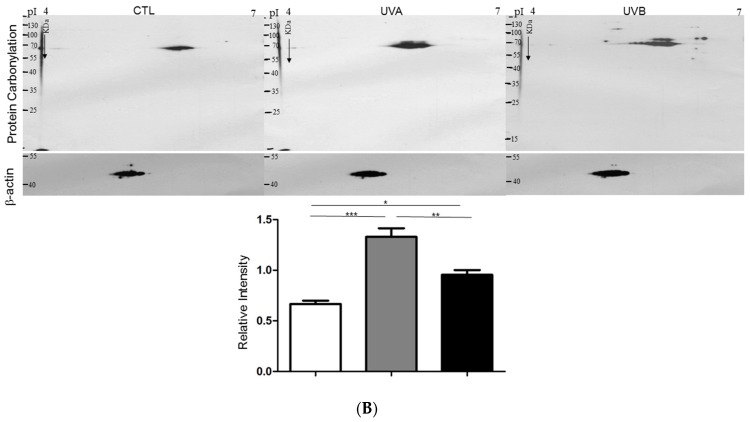
Evaluation of the oxidative stress as well as the antioxidative efficacy of raspberry extract under UVA and UVB exposure. (**A**) 8-Oxo-2’-deoxyguanosine (8-OHdG) levels were determined by immunocytochemistry and 8-OHdG-positive signal was presented as a brown color. The quantified results were indicated with the bar chart. IOD: integrated optical density. (**B**) The levels of carbonylated proteins were indicated and the quantified ratios of protein spots are performed by the bar chart. β-actin was applied as a loading control (* *p* < 0.05, ** *p* < 0.01, *** *p* < 0.001).

The enhanced production of ROS induces oxidative stress and causes oxidative DNA damage, which further results in 8-Oxo-2’-deoxyguanosine (8-OHdG) modification. To determine the oxidative stress and DNA damage potential of UV radiation, the oxidative stress was demonstrated by 8-OHdG staining. The results showed that DNA oxidation significantly increased in the dermis of the UVA-irradiated skin compared to that in the control samples, while fewer 8-OHdG positive cells were observed in the UVB-irradiated skin and mainly occurred in the epidermis (Figure 2A). Particularly, UVA exposure elicited a much higher level of 8-OHdG in the DNA compared to UVB exposure (more than two-fold). Protein carbonylation is also an important indicator of oxidative stress. Figure 2B indicates that protein carbonylation was especially induced by UVA treatment and a moderate increase in protein carbonylation was found after UVB administration in regard to the control. In addition, UVB can cause carbonylation of various proteins, while UVA application mainly leads to oxidation of the albumin. 

**Figure 3:**
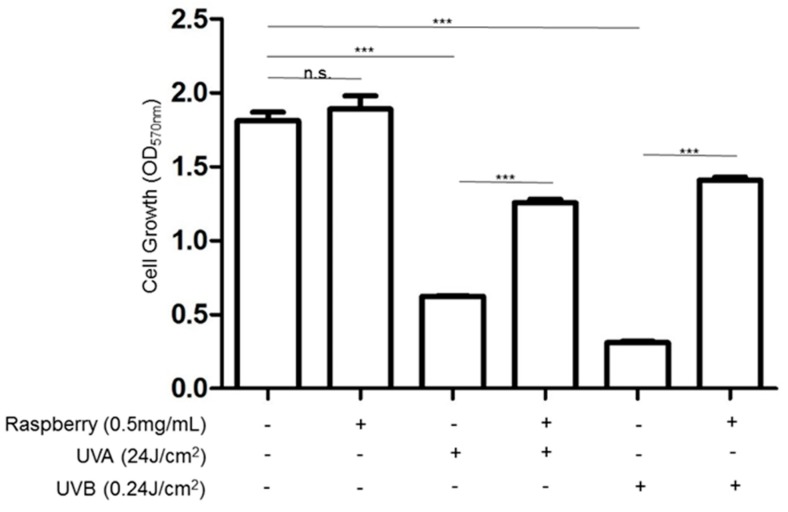
Restore the cell viability with antioxidative raspberry extract under UVA and UVB exposure. MTT assays were applied to evaluate the effects of red raspberry extract on keratinocyte (HaCaT cell) growth with or without (+ or -) the treatment of UVA and UVB. The cells were exposed to 0.5 mg/mL red raspberry extract and the data were the mean ± SD of three independent experiments. (*** *p* < 0.001; n.s., not significant.).

To further verify the cytotoxicity caused by oxidative stress due to UVA and UVB exposure, cell viability was determined by MTT assays with or without treatment of 0.5 mg/mL of raspberry extract, which has been found to contain antioxidant properties in the presence of UVA and UVB. As illustrated in Figure 3, the results showed that raspberry extract application could effectively attenuate the cell death caused by UV irradiation, suggesting that oxidative stress is a key event in UV-mediated skin cell death.

### 3.3. Effects of UVA and UVB Exposure in Cell Proliferation and Apoptosis

**Figure 4:**
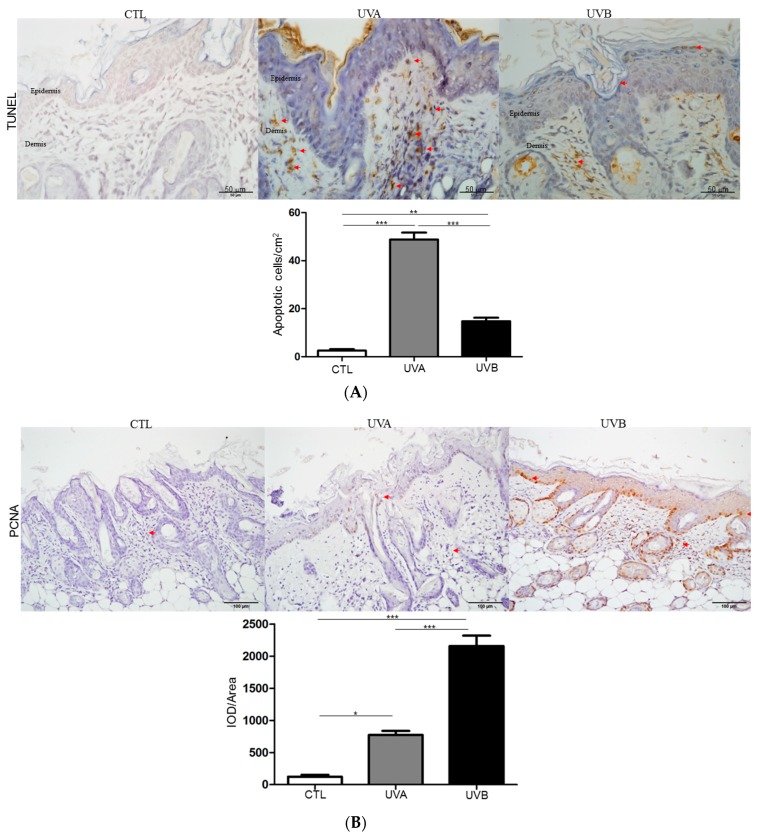
Investigation of the cell proliferation and apoptosis after treatment of UVA and UVB. (**A**) Terminal deoxynucleotidyl transferase dUTP nick end labeling (TUNEL) experiment was performed with immunohistochemical examination of nude mouse skin. The positive signal was indicated by the red arrows and the quantified results were indicated by the bar chart. (**B**) Proliferating cell nuclear antigen (PCNA) expression was examined with immunohistochemical staining. The regions with differently expressed proteins were indicated by arrows. (* *p* < 0.05; ** *p* < 0.01; *** *p* < 0.001).

Next, a TUNEL assay and PCNA expression were employed to survey the impacts on cell growth under UVA and UVB administration. As shown in Figure 4A, apoptotic cell death was evaluated by TUNEL staining. TUNEL-positive apoptotic cells rarely occurred in the control, while TUNEL-positive cells were obviously promoted in the dermis of the UVA-treated group and slightly increased in the epidermis of the UVB-applied samples, respectively. The TUNEL results were relevant to our findings of oxidative stress. On the other hand, UVB exposure significantly stimulated cell proliferation in regard to the control and the PCNA level was also markedly enhanced, whereas the UVA treatment resulted in a moderate increase in PCNA expression compared with the control, as shown in Figure 4B. As expected, these findings were consistent with the histological results.

### 3.4. Exploring Target Proteins with Proteome Tools

**Figure 5:**
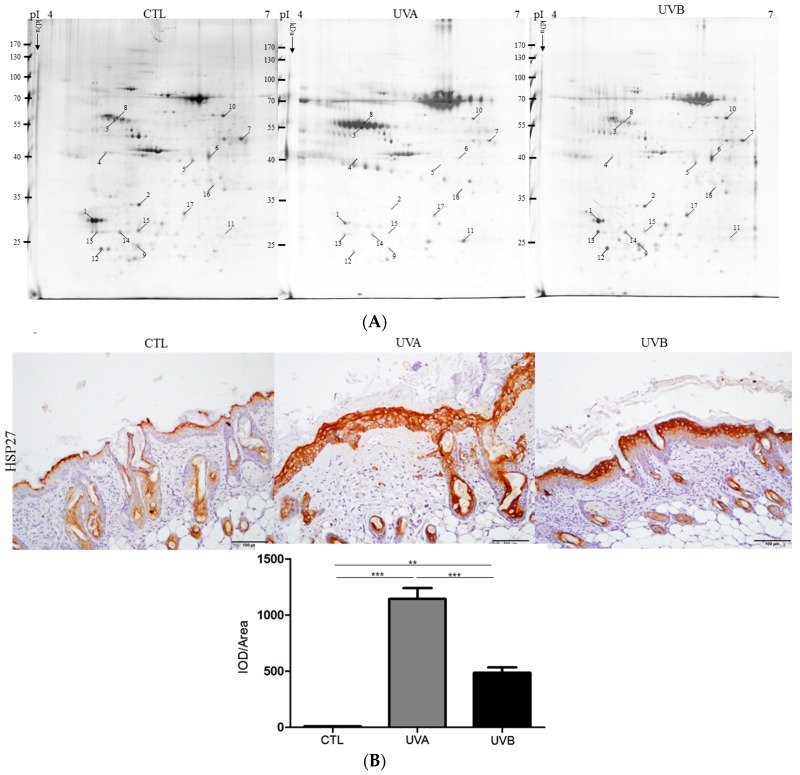

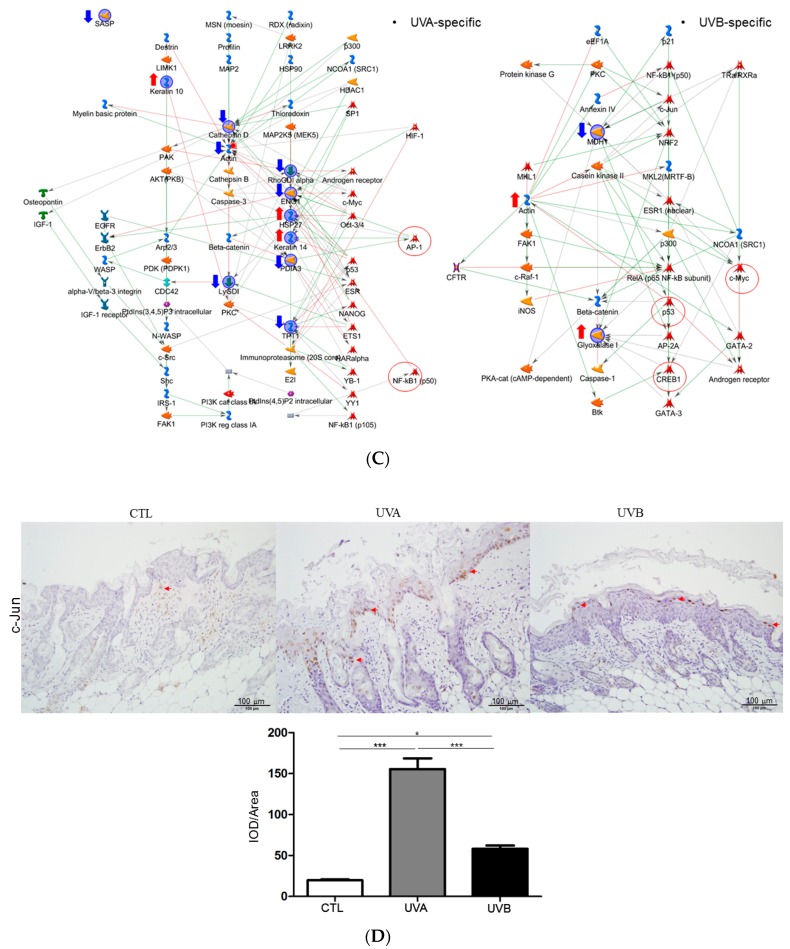

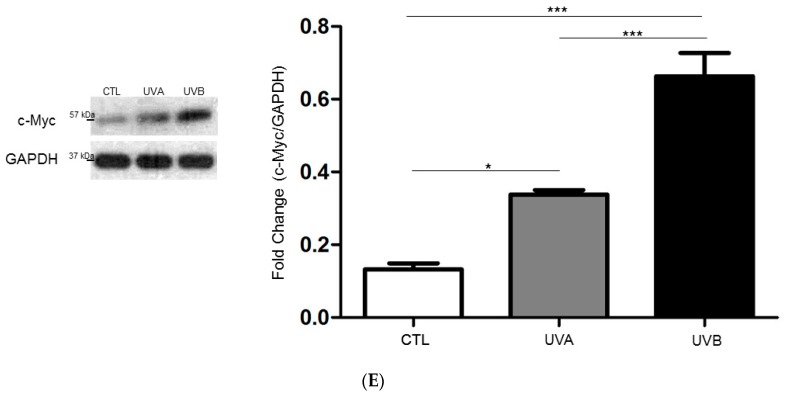
Validation of the protein changes with proteome study and biological network analysis. (**A**) Characteristic two-dimensional electrophoresis (2-DE) protein profiles of nude mouse skin. Each spot volume was determined and quantified by silver-staining (Prodigy SameSpots software). The protein spots with significant difference in volume are marked with Arabic numbers. (**B**) The expression level of HSP27 was verified by immunohistochemical experiment. The quantification of protein volume was determined with the Image Pro-Plus 4.5 computer program and presented by the bar chart. (** *p* < 0.01; *** *p* < 0.001). (**C**) Biological network analyses of differentially expressed proteins using MetaCore software. Nodes indicate proteins and lines between the nodes show direct protein–protein interplays. The different proteins on this map are represented by different symbols reflecting the functional class of the proteins. (**D**) Confirmation of the data of the c-Jun signal derived from the network analysis and the expression of c-Jun was denoted by red arrows. (**E**) The level of c-Myc was surveyed with the Western blot analysis and glyceraldehyde 3-phosphate dehydrogenase (GAPDH) was used as a loading control. The quantified results were demonstrated by the bar chart. (* *p* < 0.05; *** *p* < 0.001).

To further reveal the particular proteins and pathways related to the impact caused by various UV irradiations, 2-DE analysis was conducted to comprehensively elucidate the global protein changes. Proteins extracted from skin tissues undergoing different treatments were separated by 2-DE gels and the representative set of silver-stained gels from reproducible gel patterns of three independent experiments were demonstrated as shown in Figure 5A. Approximately 1500 protein spots appeared in each gel after 2-DE and silver staining. In-gel tryptic digestion and MS analysis unambiguously identified the 17 different proteins with significant changes in protein volume, and the results are summarized in Table 1. We then selected the meaningfully-changed proteins that played crucial roles in the skin composition and physiology for the immunohistochemistry (IHC) assay. A similar trend in the induced protein expression levels, as presented by the IHC result, was shown in the data as they appeared in the 2-DE analysis. A significant increasing tendency in HSP27 expression was characterized in the UVA-applied group in comparison to the control sample, and the UVB-exposed group showed mild upregulation in the magnitude of the HSP27 protein level with respect to the control (Figure 5B).

### 3.5. Network Analysis

MetaCore™ analytical software was used to predict the relationship of the targeted proteins revealed by proteomic analysis and the underlying mechanisms associated with the etiology after UVA and UVB administration. As shown in Figure 5C, the protein–protein interaction networks indicated that the identified proteins under UVA treatment were mainly involved in AP-1 and caspase-3 mediated inflammation as well as apoptosis, while UVB exposure predominantly led to uncontrolled proliferation triggered by oncogenes such as p53 and c-Myc. Furthermore, the expression of c-Jun was further verified with IHC, in which c-Jun was highly induced in the dermis under UVA administration while the c-Jun signal was rarely detected in the UVB-treated samples (Figure 5D). Again, the UVB exposure significantly stimulated the expression of c-Myc which was moderately increased in the UVA-applied samples with respect to the control (Figure 5E). These findings were consistent with the results from the proteome profiles and network analysis.

## 4. Discussion

Ultraviolet (UV) radiation is an environmental carcinogen and a major risk factor for skin cancer, as well as premature photoaging of the skin [28,29,30]. UVA is abundant in incoming UV radiation (90–95%) and transmits deeply into the dermis and even the hypodermis. On the other hand, UVB wavelengths, which are mainly absorbed within the epidermal layers, are a minor (5–10%), but biologically active part of UV irradiation [31]. Although proteomics has been commonly performed in various areas of the biomedical field, this technique has not been widely applied in photobiology. In the current study, we applied histological and proteome tools to highlight new insights into the impact of UVA and UVB on nude mouse skin, as numerous studies have demonstrated that UVA or UVB mediated skin responses in hairless mice are similar to those in humans.

According to the histological findings, the UVA and UVB-exposed skin showed significantly different features. Histological changes of the skin under UVA exposure showed an increase of dermis thickness and breakdown, as well as the disorganization of collagen fiber, which indicated the potential loss of skin integrity in the dermal layer. The histological result also verified the specific characteristics of the UVA-treated skin, such as inflammatory infiltration. On the other hand, the proliferation of keratinocytes without obvious alterations in the dermis was observed in the skin after UVB administration, implying that UVB could speed up the proliferative lifespan in epidermal cells [32]. Meanwhile, UV radiation-caused skin damage was closely linked to the MMPs secreted by a pool of cells from dermic-epidermic tissue [33]. UVA irradiation markedly induced MMP activity, which enhanced the degradation of collagen, leading to apoptosis of the fibroblasts and the evocation of inflammatory cells. Moreover, collagen fibers were abolished and deep furrows appeared on UVA-exposed skin. UVB exposure moderately promoted MMP activity, suggesting that UVB wavelengths will arouse minor damage in the dermis. In line with the above-mentioned results, TUNEL assays showed that UVA exposure caused the large scale apoptosis of dermal fibroblasts in comparison to the UVB-treated samples, while the level of PCNA, which is an indicator for cell proliferation, was highly upregulated in the epidermal layer under UVB irradiation, with respect to the UVA-applied group.

Reactive oxygen species (ROS) are significantly triggered by UV irradiation and are related to the mitogen-activated protein kinase (MAPK)-mediated signal pathway, which is implicated in cellular activity such as inflammatory responses as well as skin aging [34,35]. Particularly, UVA administration resulted in a higher level of DNA and protein oxidation than that of UVB, whereas UVB application stimulated a more extensive oxidative modification of proteins, implying that UVA might cause chronic damage to skin while UVB mainly leads to acute injury due to endoplasmic reticulum (ER) stress mediated by the unfolding protein response (UPR) [36]. Large amounts of ROS caused by UV exposure initiated adverse responses in the skin cells, while raspberry extract provided protection against the UV-induced death of skin cells, suggesting that oxidative stress is a principal risk in UV-mediated skin detriment.

To comprehensively reveal the protein profiles reflecting skin responses elicited by the different UV radiations, a highly-efficient 2-DE analysis and matrix-assisted laser desorption/ionization time of flight mass spectrometry (MALDI-TOF-MS) were performed. Of note, 17 protein spots displaying significant and meaningful changes were recognized, in which the protein abundance changes were highly indicative of specific cell metabolisms, including cell growth modulation, apoptosis, inflammation, protein folding, and ER stress. Selected proteins that might be pivotal to skin impacts under UVA and UVB exposure are listed as follows.

Previous reports have mentioned that HSP27 increases simultaneously with keratinocyte differentiation [37]. In this regard, HSP27 is considered to be a marker of epidermal differentiation. Our results indicated that HSP27 (spot 11) significantly increased by 4.52- and 1.86-fold in the UVA and UVB-exposed groups, respectively, showing that UVA administration is strongly implicated in the differentiation of cells compared to UVB treatment. Again, the UVB-applied skin also demonstrated a significant induction in PCNA expression levels, indicating that UVB particularly induces the proliferation of keratinocytes rather than cell differentiation.

Interestingly, serpin B5 (spot 5), which is known as a collagen modulator in protecting collagen against degradation, was significantly reduced under UVA exposure, whereas the UVB-treated samples showed a moderate decrease in protein levels, which was consistent with our previous findings that UVA administration will result in the large-scale degradation of collagen and that UVB application exhibits a minor impact on collagen breakdown.

Cathepsin D (spot 6), a novel regulator of keratinocyte growth [38], showed a remarkable decrease in the UVA-treated sample by 5.92-fold, suggesting the inhibition of epidermal cell growth, which was also in line with the results derived from histological research. Several reports have indicated that the absence of cathepsin D will promote the intracellular generation of ROS. In this regard, UVA exposure causes the decreasing expression of cathepsin D as well as an oxidative imbalance [39,40]. As expected, the oxidation of DNA and protein manifested by 8-OHdG, as well as carbonylated modification, were predominantly induced in the UVA-exposed skin, which was revealed by immunohistochemistry.

A variety of transcription factors were activated in response to the excessive presence of ROS. The network results also illustrated that UVA treatment should be primarily connected to the AP-1, NF-κB, and caspases-3-related pathways, which all induce inflammatory responses as well as apoptotic signaling cascades. On the other hand, UVB application contributes to modulation of the p53/CREB and c-Myc pathways, which are closely connected to carcinogenesis [41,42].

Several reports have also investigated the responses elicited by UV radiation, and demonstrated similar results with our findings. Lee et al. indicated that the oxidation of target peptides containing methionine (Met) residues was increased under UVA irradiation, which also reflects the extent of skin disorders [43] and the oxidative stress generated from daily exposure to external stimuli [44]. Thus, antioxidants could effectively prevent UV-caused skin apoptosis [45]. Moreover, specific molecular events were activated in response to the UV-irradiation skin including MAPK, protein kinase C (PKC), phosphoinositide 3-kinase (PI-3K), NF-κB, and AP-1 [46,47]. Although these approaches have mentioned the impact of UVA and UVB upon the same cell types or proteins, our current study particularly performed the global view for the whole skin layers under different UV irradiations with the systematic analysis, thereby, comprehensively discovering the changes in the skin microenvironment after the exposure to UVA and UVB.

**Figure 6:**
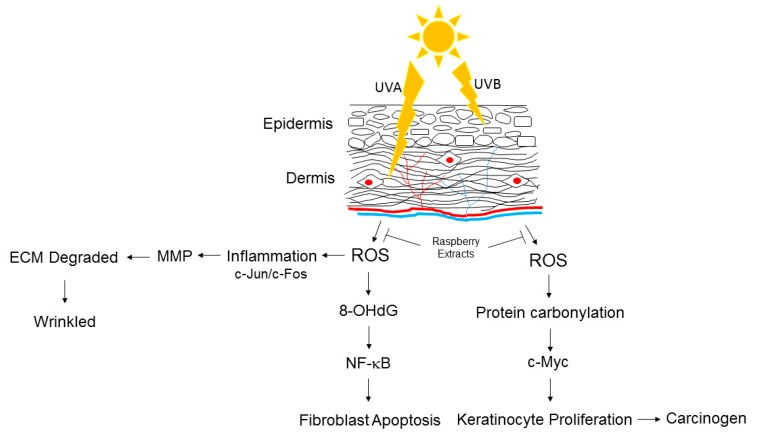
Proposed diagram of UVA- and UVB-mediated skin injury. Inducing oxidative modification of biological molecules such as protein and DNA lead to inflammatory responses, apoptotic cascades or carcinogenesis. ECM: extracellular matrix; ROS: reactive oxygen species.

Taken together, UVA strongly elicited the oxidation of biomolecules, which eventually caused apoptosis of the fibroblasts and injury to the immune cells through the caspases-3 cascade. Moreover, UVA exposure also induced inflammatory responses via activation of the AP-1 and NF-κB signaling pathways, which in turn promoted MMP expression, leading to the breakdown of collagen and elastin fibers in the dermis. On the other hand, UVB administration mainly enhanced the carbonylation of different proteins and the proliferation of keratinocytes. Meanwhile, UVB application notably induced the molecular events involved in carcinogenesis, such as p53 and c-Myc, suggesting the role of UVB in triggering skin cancer. Collectively, UVB directly causes skin carcinogenesis, while UVA induces damage to the immune system and degradation of the extracellular matrix (ECM), which facilitates the invasion and metastasis of cancer cells (Figure 6).

## 5. Conclusions

The current study provided evidence that differential UV wavelengths could trigger different changes in critical proteins and molecules that play important roles in skin metabolism and development. The application of functional proteomes and histological investigations promise a feasible method for skin studies and the evaluation of canonical mechanisms through which different UV lights mediate pathophysiologic effects on skin.

## Figures and Tables

**Table 1:** Differentially expressed proteins in the nude mice skin under UVA- and UVB-inducing.

Spot No.	Protein	Accession No.	Mw (kDa)	pI	Matched- Peptides	SCORE ^(a)^ (Sequence Coverage %)	Ratios ^(b)^			Biological Function
							UVA/CTL	UVB/CTL	*p*-Value ^(c)^	
1	Stratifin (1433S)	O70456	27.803	4.72	12	101 (48%)	−6.62 ± 0.07	−0.92 ± 0.04	0.002	Involved in the regulation of both general and specialized signaling pathways. It also controls protein synthesis and epithelial cell growth via protein kinase B/mammalian target of rapamycin (Akt/mTOR) pathway.
2	Skin aspartic protease (SASP)	Q09PK2	33.637	5.07	11	65 (24%)	−5.84 ± 0.02	−0.32 ± 0.06	0.037	Majorly expresses in the epidermis and hair follicles.
3	Cytokeratin-14 (K1C14)	Q61781	53.176	5.10	37	329 (71%)	7.67 ± 0.02	−0.57 ± 0.02	0.005	Enhances KRT5-KRT14 filaments to self-organize into large bundles and promotes the properties related to resilience of keratin intermediate filaments.
4	40S Ribosomal protein SA (RSSA)	P14206	32.935	4.80	10	90 (34%)	−1.89 ± 0.02	−0.57 ± 0.04	0.055	Involved in cell adhesion to the basement membrane and activation of signaling transduction cascades.
5	Serpin B5/Maspin (SPB5)	P70124	42.484	5.55	18	195 (66%)	−2.96 ± 0.06	−0.86 ± 0.01	0.037	Inhibits the growth, invasion, and metastatic properties of mammary tumors.
6	Cathepsin D (CATD)	P18242	45.381	6.71	13	134 (31%)	−5.92 ± 0.01	0.55 ± 0.02	0.049	Regulation in intracellular protein breakdown.
7	α-enolase (ENOA)	P17182	47.322	6.7	24	231 (58%)	−3.18 ± 0.04	−1.53 ± 0.01	0.042	Multifunctional enzyme to play a role in various processes including growth control, hypoxia tolerance, and allergic responses.
8	Cytokeratin-10 (K1C10)	P02535	57.178	5.00	12	71 (19%)	6.52 ± 0.03	−2.86 ± 0.02	0.008	Establishment of the epidermal barrier on skin.
9	Lactoylglutathione lyase (LGUL)	Q9CPU0	20.967	5.24	13	139 (55%)	−1.59 ± 0.01	1.64 ± 0.02	0.003	Regulation of tumor necrosis factor (TNF)-mediated activation of NF-kappa-B.
10	Protein disulfide-isomerase A3 (PDIA3)	P27773	57.099	5.88	27	260 (50%)	−2.23 ± 0.04	−0.28 ± 0.02	0.084	Induces the rearrangement of -S–S- bonds in proteins.
11	Heat shock 27 kDa protein (HSP27)	P14602	23.057	6.12	7	77 (35%)	4.52 ± 0.03	1.86 ± 0.02	0.014	Functions as a molecular chaperone to maintain denatured proteins in a folding-competent state.
12	Translationally-controlled tumor protein (TCTP)	P63028	19.592	4.72	12	103 (44%)	−2.52 ± 0.06	0.56 ± 0.02	0.038	Calcium binding and microtubule stabilization.
13	Proteasome subunit alpha type-5 (PSA5)	Q9Z2U1	26.565	4.74	11	104 (56%)	−1.52 ± 0.03	2.86 ± 0.02	0.004	Component of the 20S core proteasome complex linked to the degradation of intracellular proteins.
14	Rho GDP-dissociation inhibitor 2 (GDIR2)	Q61599	22.894	4.97	9	98 (69%)	−0.32 ± 0.13	0.86 ± 0.46	0.024	Involved in reorganization of the actin cytoskeleton through Rho family members.
15	Rho GDP-dissociation inhibitor 1 (GDIR1)	Q99PT1	23.450	5.12	14	133 (59%)	−1.36 ± 0.08	−0.56 ± 0.02	0.005	Modulates Rho proteins homeostasis.
16	60S acidic ribosomal protein P0 (RLA0)	P14869	34.366	5.91	8	79 (41%)	−0.38± 0.03	−0.58±0.04	0.086	Playing a pivotal role in the interaction of the ribosome with GTP-bound translation factors.
17	Beta-actin (ACTB; Frag.)	P60710	42.052	5.29	7	85 (47%)	−0.89 ± 0.01	1.64 ± 0.02	0.004	Actin exists in both monomeric (G-actin) and polymeric (F-actin) forms to regulate functions including cell motility and contraction. It also localizes in the nucleus to control gene transcription, motility and repair of damaged DNA.

(a) Mouse NCBIprot 20180429 (152462470 sequences; 55858910152 residues); (b) Ratios to control demonstrated the fold changes of protein volume between UVA- and UVB-exposed subjects versus control samples, respectively. “+” meant upregulation and “−” indicated downregulation of protein volume; (c) *p*-values were produced by analyzing the gel images with Prodigy SameSpots^TM^ software. *p* < 0.05 was considered significant for the differences.

## Abbreviations

2-DE	two-dimensional electrophoresis
DNP	2,4-dinitrophenylhydrazine
MALDI-TOF-MS	matrix-assisted laser desorption/ionization time of flight mass spectrometry
PMF	peptide mass fingerprinting
ROS	reactive oxygen species
IPG	immobilized pH gradient
GAPDH	glyceraldehyde 3-phosphate dehydrogenase
IHC	immunohistochemistry
MAPK	mitogen-activated protein kinase
DTT	dithioerythritol
SDS-PAGE	sodium dodecyl sulfate-polyacrylamide gel electrophoresis
UV	ultraviolet
8-OHdG	8-Oxo-2’-deoxyguanosine
TBST	tris-buffered saline Tween-20
PVDF	polyvinylidene difluoride
TUNEL	terminal deoxynucleotidyl transferase dUTP nick end labeling
ECM	extracellular matrix
UPR	unfolding protein response
MMP	matrix metallopeptidases
PCNA	proliferating cell nuclear antigen
